# Mapping combined with principal component analysis identifies excellent lines with increased rice quality

**DOI:** 10.1038/s41598-022-09976-2

**Published:** 2022-04-08

**Authors:** Qi Wang, Xiaonan Li, Hongwei Chen, Feng Wang, Zilong Li, Jiacheng Zuo, Mingqian Fan, Bingbing Luo, Pulin Feng, Jiayu Wang

**Affiliations:** Rice Research Institute of Shenyang Agricultural University/Key Laboratory of Rice Biology & Genetic Breeding in Northeast China (Ministry of Agriculture and Rural Areas), Shenyang, 110866 People’s Republic of China

**Keywords:** Plant molecular biology, Plant breeding

## Abstract

Quality-related traits are some of the most important traits in rice, and screening and breeding rice lines with excellent quality are common ways for breeders to improve the quality of rice. In this study, we used 151 recombinant inbred lines (RILs) obtained by crossing the northern cultivated japonica rice variety ShenNong265 (SN265) with the southern indica rice variety LuHui99 (LH99) and simplified 18 common rice quality-related traits into 8 independent principal components (PCs) by principal component analysis (PCA). These PCs included peak and hot paste viscosity, chalky grain percentage and chalkiness degree, brown and milled rice recovery, width length rate, cooked taste score, head rice recovery, milled rice width, and cooked comprehensive score factors. Based on the weight ratio of each PC score, the RILs were classified into five types from excellent to poor, and five excellent lines were identified. Compared with SN265, these 5 lines showed better performance regarding the chalky grain percentage and chalkiness degree factor. Moreover, we performed QTL localization on the RIL population and identified 94 QTLs for quality-related traits that formed 6 QTL clusters. In future research, by combining these QTL mapping results, we will be using backcrossing to aggregate excellent traits and achieve quality improvement of SN265.

## Introduction

Rice is one of the most important food crops, with more than half of the global population dependent on it as a staple food^1^. In recent years, with the increasing demand for high-quality rice, the quality-related traits of rice have gained increasing worldwide attention, and the improvement and enhancement of rice quality-related traits will help provide greater returns on investment for smallholder farmers,therefore, breeding new varieties of high-quality rice to meet market demand has become one of the main initiatives for rice breeders and molecular geneticists and a key objectives of rice research^2,3^.

Rice quality-related traits broadly include processing quality, appearance quality, cooking quality, and eating quality^4^. The processing quality of milled rice refers to the ability of the grains to withstand challenge and polishing without breaking, and it determines the final yield of edible rice^5^. Appearance quality directly defines the market value of rice and is closely related to grain yield and head rice production^6^. The cooking and eating quality of food affects the sensory perception of people during meals, and high-quality rice should be light, oily, slightly sweet, strong, soft, and sticky after steaming^7^.

Quality-related traits often require the measurement of many indicators, and it is difficult for breeders to balance these indicators because of the complex correlation between them^8^. Principal component analysis (PCA) is a method for simplifying datasets containing highly relevant and relatively complex information by extracting as much key information as possible from original information^9,10^. In previous rice studies, the use of PCA to simplify multiple agronomic trait indicators into principal components (PCs) as quantitative indicators has been validated^11^.

Quality-related traits are considered quantitative traits controlled by multiple genes. To date, many QTLs for quality-related traits have been identified by different researchers. Yun et al.^12^, Gao et al.^13^, Ponce et al.^14^, Qiu et al.^15^, and Arikit et al.^16^ identified QTLs for different quality-related traits using different genetic populations. However, it is difficult to use these QTLs to screen excellent lines and assess quality improvement given the many quality-related trait indicators and lines.

In this study, we used RILs constructed from a cross between the northern elite japonica cultivar SN265 and the southern indica cultivar LH99 as materials to perform PCA and QTL mapping of 18 rice quality-related traits, and we identified five lines with excellent quality. Based on the results of genetic and QTL mapping, these lines with excellent quality can be used as intermediate materials to achieve quality improvement of SN265.

## Results

### Phenotypic variation of the parents

**Figure 1:**
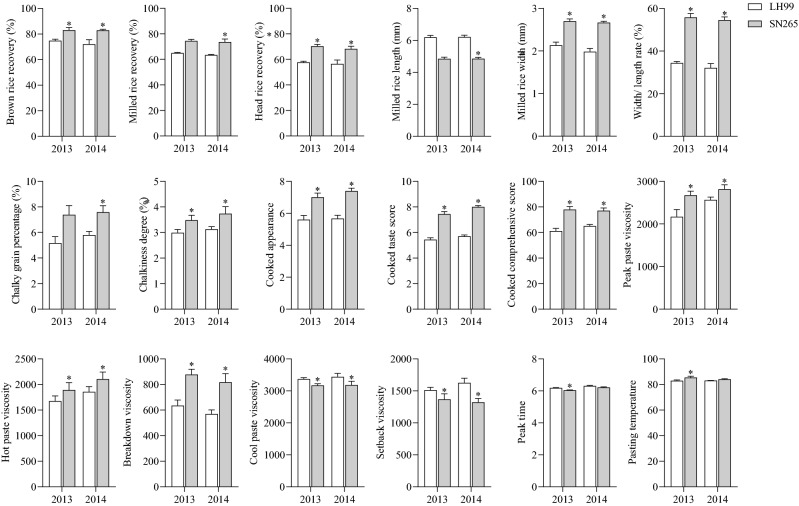
Phenotypes of quality traits between SN265 and LH99. * represents significant at *P* < 5% (Student’s t-test).

There were significant differences in quality-related traits between SN265 and LH99 across both years (S1, Fig. 1). In terms of milling quality, SN265 had a higher BRR, MRR, and HRR than LH99. With regard to appearance quality, SN265 showed short round milled rice and high chalkiness, while with respect to eating quality, SN265 showed superior CA, CTS, and CCS. In the RVA spectral eigenvalues, SN265 exhibited a higher PKV, HPV, and BKV than LH99, a lower CPV and SBV than LH99, and similar PeT and PaT values as LH99.

### Phenotypic variation in the recombinant inbred lines (RILs)

**Figure 2:**
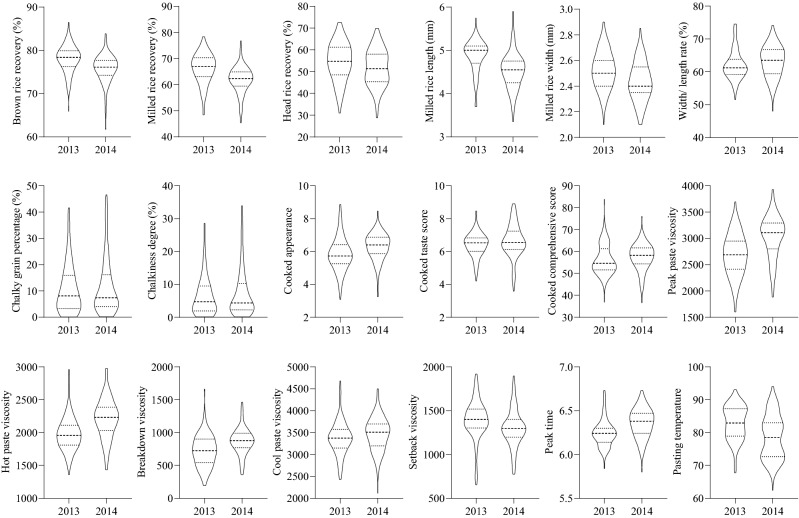
Distribution of quality traits in the RILs population.

Quality-related traits in both years were slightly different, but the general trend was consistent. The recombinant inbred lines (RILs) differed considerably, with an approximately normal distribution overall and bidirectional transgressive segregation (S1, Fig. 2); these results suggest that these genetic characteristics involve quantitative traits, in line with QTL mapping requirements.

### Correlation analysis between quality-related traits

**Figure 3:**
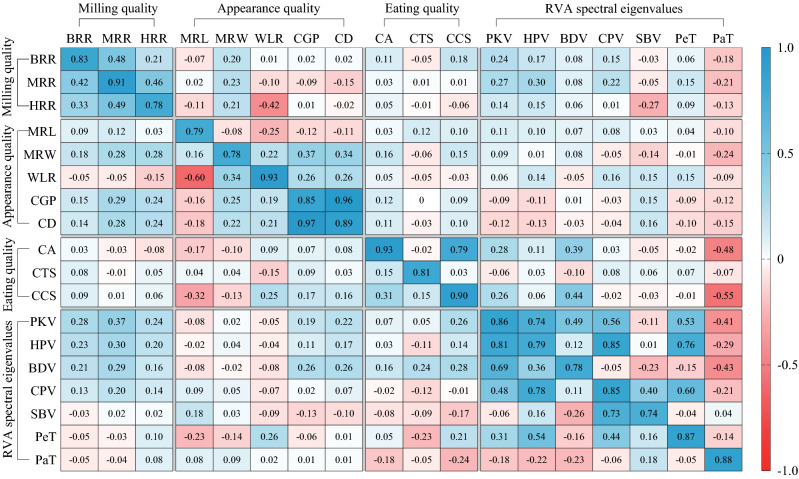
Pearson correlation coefficient to analyze the relevance on RILs Quality-related traits. The lower triangle indicates the correlation coefficient for 2013, the upper triangle indicates the correlation coefficient for 2014, and the diagonal data indicates the correlation coefficient between two years for the same trait.

There is a general correlation between the various indicators of quality-related traits. The RILs exhibited excellent repeatability of the same quality-related traits across both years, with some correlations occurring between different quality-related traits (Fig. 3).

### PCA of the RIL population

In PCA, a dataset is considered to be representative when the cumulative proportion of variance of the principal components is greater than 80%. In this study, the eigenvalues of the top 8 principal components were all greater than 0.87, with proportions of variance of 22.09, 13.31, 12.46, 10.97, 6.41, 5.53, 5.19 and 4.84%. Their cumulative proportion of variance reached 80.80% (S2), meaning that the top 8 independent principal components represented 80.80% of the variation in the 18 quality-related traits.

**Figure 4:**
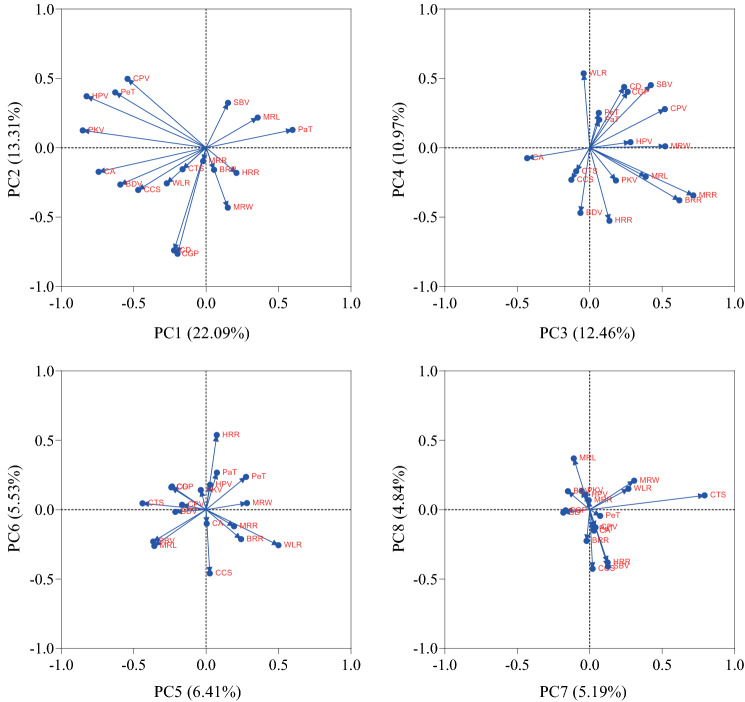
Loadings of top 8 principal components.

The first principal component was characterized by higher positive loading for PaT (0.597) and higher negative loading for CA (−0.742), CCS (−0.469), PKV (−0.851), HPV (−0.825), BDV (−0.592), CPV (−0.542) and PeT (− 0.627); this PC is referred to as the peak and hot paste viscosity factor. The second principal component had a higher positive loading for CPV (0.496) and higher negative loadings for MRW (−0.432), CGP (−0.764), and CD (−0.740); this PC is referred to as chalky grain percentage and chalkiness degree; The third principal component was characterized by higher positive loadings for BRR (0.619), MRR (0.715), MRW (0.521), CPV (0.519), and SBV (0.422) and a higher negative loading for CA (−0.432); this PC is referred to as the brown and milled rice recovery factor. The fourth principal component had higher positive loadings for WLR (0.535), CGP (0.403), CD (0.438), and SBV (0.451) and higher negative loadings for HRR (−0.527), and BDV (−0.470); this PC is referred to as the width length rate factor. The fifth principal component had a higher positive loading for WLR (0.500) and higher negative loading for CTS (−0.439); this PC is referred to as the cooked taste score factor; The sixth principal component had a higher positive loading for HRR (0.538) and a higher negative loading for CCS (−0.459); this PC referred to as the head rice recovery factor. The seventh principal component had higher positive loadings for CTS (0.793) and MRW (0.307); it is referred to as the milled rice width factor. The eighth principal component had higher negative loadings for CCS (−0.426) and SBV (−0.410); it is referred to as the cooked comprehensive score factor (S2, Fig. 4).

### QTL analysis of rice processing quality

**Figure 5:**
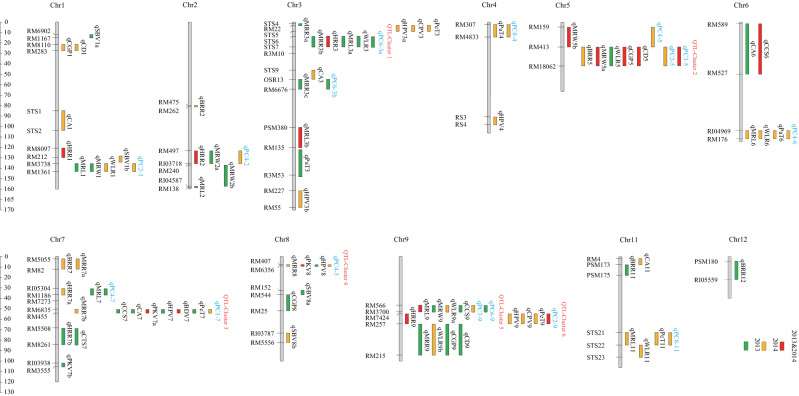
Location on the genetic map of QTL loci detected for quality traits. Green indicates QTL loci detected in 2013, yellow indicates QTL loci detected in 2014 and red indicates QTL loci detected in both years.

Eighteen rice milling quality QTLs were detected in both years, on chromosomes 1, 2, 3, 5, 7, 8, 9, 11, and 12, with LOD values ranging from 2.00 to 4.73 and individual QTL contribution rates ranging from 4.79 to 15.56%. *qHRR1*, *qHRR2*, *qHRR3* and *qHRR9* were reproducibly detected over two years. Of these, the enhancing allele of *qHRR1* was from LH99, and the enhancing alleles of *qHRR2*, *qHRR3*, and *qHRR9* were from SN265 (S3, Fig. 5).

### QTL analysis of rice appearance quality

A total of 28 QTLs for rice appearance quality were detected in both years on chromosomes 1, 2, 3, 5, 6, 7, 8, 9, 11 and 12. The LOD values ranged from 2.01 to 11.00, and the individual QTL contribution rates ranged from 4.08 to 32.59%. Six QTLs (*qMRL9*, *qMRW5a*, *qMRW5b*, *qWLR6*; *qCGP5*, and *qCD5*) were reproducibly detected across both years, including one QTL for grain length—*qMRL9*; two QTLs for grain width—*qMRW5a* and *qMRW5b*; one QTL for aspect ratio—*qWLR6*; one QTL for chalky grain percentage—*qCGP5* and one QTL for chalkiness degree—*qCD5*. The quality-enhancing alleles of *qMRL9* and *qMRW5b* were from SN265, and those of *qMRW5a*, *qWLR6*, *qCGP5*, and *qCD5* were from LH99 (S3, Fig. 5).

### QTL analysis for cooking and eating quality

Nine QTLs for rice cooking and eating quality were detected in both years on chromosomes 1, 3, 6, 7, 9 and 11. The LOD values ranged from 2.00 to 3.32, and the individual QTL contributions ranged from 6.62 to 26.03%. One QTL, *qCS6*, was detected in both environments, and the enhancing allele was from SN265 (S3, Fig. 5).

### QTL analysis for RVA

A total of 23 QTLs, associated with characteristic values of rice RVA profiles were detected in both years on chromosomes 1, 3, 4, 6, 7, 8, 9, and 11. The LOD values ranged from 2.00 to 4.21, and the individual QTL contributions ranged from 3.22 to 13.78%. Three QTLs (*qPKV7*, *qPKV8*, and *qBDV7*) were detected in both years, including two peak paste viscosity QTLs (*qPKV7* and *qPKV8*) and one breakdown viscosity QTL (*qBDV7*). The quality-enhancing alleles of *qPKV7*and *qBDV7* were from SN265, and that of *qPKV8* was from LH99 (S3, Fig. 5).

### QTL analysis for PC

A total of 16 QTLs associated with rice PC scores were detected in both years on chromosomes 1, 2, 3, 4, 5, 6, 7, 9 and 11. The LOD values ranged from 2.68 to 5.18, and the individual QTL contributions ranged from 7.58 to 16. 62%. Two QTLs were detected in both years (*qPC2-9* and *qPC3-5*), with PC2 having higher chalkiness degree and chalky grain percentage values and PC3 having higher cool paste viscosity and setback viscosity values. The quality-enhancing alleles of *qPC2-9* and *qPC3-5* were from LH99 (S3, Fig. 5).

### Multiple QTLs are distributed in clusters

A QTL cluster is defined as an interval on a chromosome containing several QTLs, usually with cumulative effects, or one pleiotropic QTL with major effects. In this study, 59 QTLs were distributed in clusters on chromosomes 1, 2, 3, 5, 6, 7, 8, 9, and 11, accounting for 62.76% of the total number of QTLs. Six QTL clusters containing OTLs detected by PCA across both years were identified for the quality-related traits (Fig. 5). QTL cluster 1 was between the molecular markers STS6 and STS7 on chromosome 3 and covered a genetic distance of approximately 7.1 cM; this cluster contained *qMRR3b*, *qHRR3*, *qMRL3a*, *qWLR3* and *qPC6-3a*. QTL-cluster 2 was between molecular markers RM413 and RM18062 on chromosome 5 and covered a genetic distance of approximately 12.8 cM; this cluster contained *qBRR5*, *qMRW5a*, *qWLR5*, *qCGP5*, *qCD5*, *qPC2-5* and *qPC3-5*. QTL cluster 3 was between molecular markers RM6835 and RM455 on chromosome 7 and covered a genetic distance of approximately 4.9 cM; this cluster contained *qMRR7b*, *qCCS7*, *qCA7*, *qPKV7a*, *qHPV7*, *qBDV7*, *qPeT7* and *qPC1-7*. QTL-cluster 4 was between molecular markers RM407 and RM6356 on chromosome 8 and covered a genetic distance of approximately 3.4 cM; this cluster contained *qMRR8*, *qPKV8*, *qHPV8* and *qPC4-3*. QTL cluster 5 was between molecular markers RM566 and RM3700 on chromosome 9 and covered a genetic distance of approximately 4.6 cM; this cluster contained *qMRL9*, *qMRW9*, *qWLR9a*, *qCCS9*, *qPC3-9* and *qPC6-9*. QTL cluster 6 was between molecular markers RM7424 and RM257 on chromosome 8 and covered a genetic distance of approximately 5.4 cM; this cluster contained *qHRR9*, *qHPV9*, *qCPV9*, *qPeT9* and *qPC2-9*.

### Selection and genetic analysis of excellent lines

**Figure 6:**
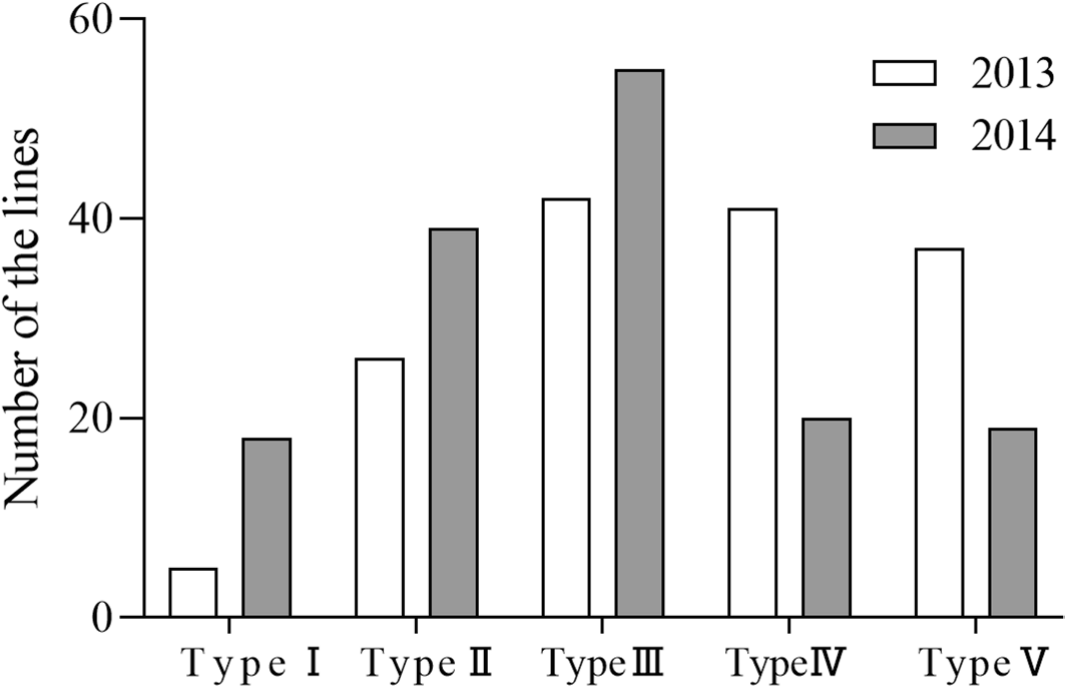
Distribution of total score in the RILs population. TypeI, TypeII, TypeIII, TypeIV and TypeV correspond to number of the lines with total score in the interval n > 1.00, 1.00 ≥ n > 0.50, 0.50 ≥ n > 0.00, 0.00 ≥ n > -0.50 and n ≤ -0.50.

**Figure 7:**
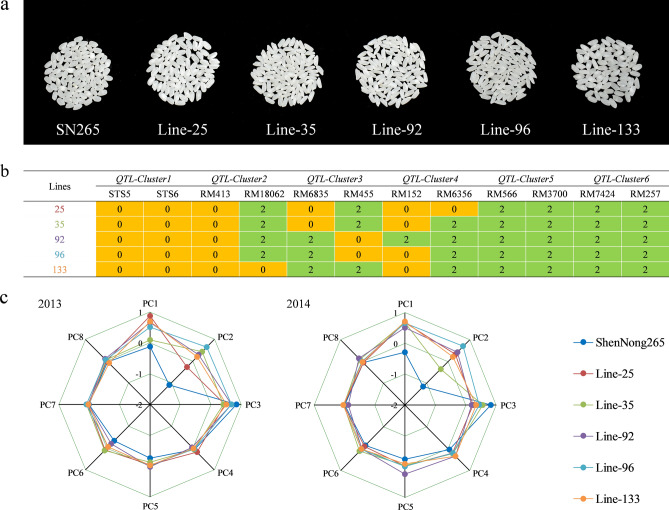
(**a**) Photographs of head rice from SN265 and five excellent quality lines. (**b**) Genetic performance of five excellent quality lines on six QTL clusters, where 0 represents inheritance from SN265 and 2 represents inheritance from LH99. (**c**) Comparison of the scores of SN265 and the five excellent quality lines on the eight principal components.

The RILs were grouped together according to their weights based on the scores of the 8 principal components, with scores ranging from −1.67 to 2.07. The lines were classified according to their total scores and were divided into 5 types, which were ranked in order from excellent to poor based on the quality-related traits (Fig. 6). Five lines were in the top group in both years (Fig. 7a). The PC1 and PC2 values of these 5 lines were significantly higher than those of SN265 in both years, among which PC2 showed a larger increase, while the values for the other 6 principal components were not significantly different from those of SN265 (Fig. 7c). PC1 mainly regulates the peak paste viscosity and hot paste viscosity factor, and PC2 mainly regulates the chalkiness degree and chalky grain percentage. The Five excellent rice lines carrying six QTL clusters were analyzed (Fig. 7b). Genetic regions including QTL cluster 1 were found in 5 lines, while those for QTL cluster 2 were found in line-133 and QTL cluster 3 in line-25; all regions were from SN265, indicating that the QTLs contributing to the quality-related traits in these lines were all from SN265. Therefore, these QTL clusters in these specific lines could not be used for the genetic improvement of SN265. The genetic regions including QTL cluster 4 and QTL cluster 5 were found in 5 lines and those for cluster 3 were found in line-92; all regions were from LH99, indicating that the QTLs contributing to the quality-related traits in these lines were all from LH99. Therefore, these QTL clusters in the abovementioned lines could be used for the genetic improvement of quality in SN265. Notably, the QTL for PC2, *qPC2-9*, which was detected in both years, was located in QTL cluster 6. The chalkiness degree and chalky grain percentage of the five lines were significantly lower than those of SN265 and close to those of LH99 (S4). The region containing QTL cluster 6 was derived from LH99, which supported the accurate identification of *qPC2-9* to some extent. Therefore, *qPC2-9* has significant potential for improving the quality of SN265.

## Discussion

### QTL clusters contribute to the mining of genes for quality-related traits

Quality improvement is one of the most important ways to adapt rice to the consumer market^17^. With the advancement of science and technology, mining new QTLs for quality-related traits, cloning relevant genes and applying quality improvement to actual production in combination with molecular marker-assisted breeding and transgenic and gene editing technologies is a feasible way to improve the quality of rice^2^. The genetic basis for quality-related traits in rice is complex, and QTLs acting on multiple traits in the same chromosomal region are common^18^. Ponce et al.^14^ used a multiparent advanced generation cross-population to localize QTLs for rice cooking and eating quality and identified 17 QTLs, of which 9 were in clusters that contained *GSSI* genes and 5 were in clusters that contained *SSIIa* genes. These QTLs and the markers highly associated with their underlying traits will be useful for breeding indica rice to improve cooking and eating quality. Yao et al.^19^ measured RVA and mapped 93 QTLs in four environments based on 151 RILs. These QTLs formed five clusters on chromosomes 1, 3, 6, 7, and 11, and the study found that AC and RVA traits were not influenced by indica-japonica subspecies differentiation, indicating that the RVA curve was mainly influenced by the *Wx* gene.

In this study, we identified 94 QTLs for quality-related traits, of which 59 QTLs were distributed in six different clusters. Compared with previous studies, the QTLs we identified quality-related traits partially corresponded to those found by Yao et al.^19^, Yan et al.^20^, and Nelson et al.^21^. Among them, the gene *GW5*, which significantly affects grain width and grain weight in rice, has been cloned within the interval of QTL cluster 2^22^. Within the interval of QTL cluster 3 major quantitative trait locus, *GLW7*, which encodes the plant-specific transcription factor *OsSPL13*, was reported to positively regulate cell size in the grain hull, resulting in enhanced rice grain length and yield^23^. QTL cluster 4 contains the cloned gene *OsSSIIIa*, which affects the structure of amylopectin, amylase content, and physicochemical properties of starch granules^24^. *GS9* is a cloned gene that has been found to regulate grain length and reduce grain chalkiness in rice; this gene is located within the interval of QTL cluster 6^25^. In the intervals of QTL-cluster 1 and QTL-cluster 5, we found no genes for rice quality-related traits that have been cloned at present,however, these two intervals should be explored further.

### Selection and identification of rice lines with high quality

Quality-related traits are complex traits that are influenced by a combination of multiple factors and genes and are susceptible to external environmental conditions, making it difficult to fully and truly evaluate the quality of rice with a single indicator^26,27^. PCA is a statistical analysis method that converts many indicators into a few composite indicators, making complex problems simple and their analysis intuitive through dimensionality reduction^9,10^. In this study, 18 rice quality indicators were simplified into 8 mutually independent principal components using PCA that better reflected the basic characteristics of rice quality indicators; the cumulative contribution of all PCs was 80.80%.

Stable RILs are valuable breeding resources for studying rice quality. On the one hand, the genetic background of RILs is clear and can be used for gene mapping, and on the other hand, the quality of RILs shows a normal distribution with bidirectional transgressive segregation, thus screening lines with high affinity and excellent quality could prevent the use of intermediate materials when aggregating excellent traits^28^. In this study, we calculated a total score by weighting the 151 RILs base on 8 principal components and classified them into 5 types from superior to inferior, 5 lines with excellent quality were selected. Compared with SN265, these 5 lines showed better performance regarding chalky grain percentage and chalkiness degree factor. In future research, we can use the genetic map and the traits identified in excellent lines to achieve quality improvement in SN265 backcrosses.

## Materials and methods

### Plant materials and cultivation

We established a population of 151 lines of isolated RILs (F_8_) by crossing the elite japonica cultivar SN265 with the indica cultivar LH99. The rice plants were grown in 2013 and 2014 in experimental fields at Shenyang Agricultural University with conventional water and fertilizer management. After maturity, the rice was threshed by plant row and the threshed seeds were kept in a cool and ventilated place for 3 months to measure their quality.

### Measurement of rice quality

Traits measured in this experiment include milling, appearance, cooking and eating quality and RVA spectral eigenvalues. The experiment was conducted with three biological repetitions and t tests were performed for statistical analysis.

The milling quality includes brown rice recovery (BRR), milled rice recovery (MRR) and head rice recovery (HRR). The calculations are as follows:

BRR (%) = Weight of brown rice/Weight of rough rice × 100.

MRR (%) = Weight of total milled rice/Weight of rough rice × 100.

HRR (%) = Weight of head rice/Weight of rough rice × 100.

Appearance quality includes milled rice length (MRL), milled rice width (MRW), width length rate (WLR), chalky grain percentage (CGP) and chalkiness degree (CD). These traits were measured by an ES-1000 rice quality analyser^29^.

Cooking and eating quality includes cooked appearance (CA), cooked taste score (CTS), and cooked comprehensive score (CCS). The milled rice grains were washed and placed in distilled water in a 1:1.2 proportion for 1 h, followed by steaming them for 30 min, maintenance at that temperature for 10 min, and even stirring in the fume cabinet for 20 min. The rice grains were measured with a rice taste meter (SATAKE-STA1B) after 2 h at room temperature.

The RVA spectral eigenvalues, including peak paste viscosity (PKV), hot paste viscosity (HPV), breakdown viscosity (BDV), cool paste viscosity (CPV), setback viscosity (SBV), peak time (PeT) and pasting temperature (PaT), were measured by the American Association of Cereal Chemists Standard Method (AACC 61‐02) (1995) as described by Bao et al.^30^.

### Principal component analysis (PCA)

Principal component analysis of the 18 quality-related traits in rice was performed using GraphPad Prism 9 software, with standardized method selection, and principal components were selected based on eigenvalues. The eigenvalues (E), proportions of variance (PV), cumulative proportions of variance (CPV), loadings and PC scores were obtained. Among them, PC scores were used for QTL mapping. The total score was calculated according to the following formula. In the formula, ${W}_{i}$ is the weight of the ith principal component, ${P}_{i}$ is the proportion of variance of the ith principal component, ${T}_{i}$ is the total score of the ith lines and *Si* is the PC scores of the ith lines.

$${W}_{i}={P}_{i}/{\sum }_{i=n}^{n}{P}_{i} (i=\mathrm{1,2},3,\dots ,n)$$

$${T}_{i}={\sum }_{i=n}^{n}\left({W}_{i}\times {S}_{i}\right) (i=\mathrm{1,2},3,\dots ,n)$$

### Genetic and QTL mapping analysis

We used 144 polymorphic simple sequence repeat (SSR) and insertion/deletion (Indel) markers^31,32^ distributed among 12 chromosomes to establish a genetic map. The QTL analysis was based on inclusive composite interval mapping implemented by QTL IciMapping 4.0^33^. The QTLs were named according to the guidelines described by McCouch^34^.

### Consent for publication

All authors and associated institutes have consented to the publication of this work.

### Compliance statements

The experimental studies conducted and the plant materials used in this paper comply with relevant institutional, national, and international guidelines and legislation.

## Supplementary Information

Supplementary Information.

## Data Availability

All data generated or analyzed during this study are included in this published article and its Supplementary Information files.
