# Supplementary material for: Mapping combined with principal component analysis identifies excellent lines with increased rice quality
Source: Sci Rep. 2022 Apr 8;12:5969. doi: 10.1038/s41598-022-09976-2 (PMC8993813; doi:10.1038/s41598-022-09976-2)
Supplement: Supplementary file 1 — Supplementary Information. [file 41598_2022_9976_MOESM1_ESM.pdf]

Mapping combined with principal component analysis identifies excellent lines with increased rice quality

Qi Wang[1], Xiaonan Li[1], Hongwei Chen[1], Feng Wang[1], Zilong Li[1], Jiacheng Zuo[1], Mingqian Fan[1], Bingbing Luo[1], Pulin Feng[1], Jiayu Wang[1*]

S1. Distribution of rice quality traits in parental and RILs populations, values are means ± SDs of three biological replicates.

Traits	Years	Parents		RILs			
		LH99	SN265	Mean ± SD	Skewness	Kurtosis	Range
BRR(%)	2013	74.75 ± 1.20	83.05 ± 2.05	78.02 ± 3.19	-0.52	1.05	65.88 - 86.44
	2014	72.00 ± 3.53	82.95 ± 0.77	75.81 ± 3.21	-1.11	2.64	61.73 - 83.87
MRR(%)	2013	65.00 ± 0.43	74.50 ± 1.15	66.24 ± 5.75	-0.69	0.67	42.90 – 82.99
	2014	63.43 ± 0.47	73.63 ± 2.28	61.91 ± 5.21	-0.61	0.77	44.43 - 76.80
HRR(%)	2013	57.69 ± 0.83	70.29 ± 1.50	54.51 ± 8.76	-0.53	-0.47	24.00 - 76.10
	2014	56.46 ± 3.01	68.22 ± 2.08	51.63 ± 8.25	-0.32	-0.33	21.80 – 75.20
MRL(mm)	2013	6.19 ± 0.12	4.85 ± 0.10	4.86 ± 0.37	-1.17	1.55	3.70 - 5.75
	2014	6.22 ± 0.10	4.86 ± 0.07	4.52 ± 0.41	0.16	0.95	3.35 - 5.90
MRW(mm)	2013	2.13 ± 0.07	2.70 ± 0.05	2.52 ± 0.15	0.01	-0.13	2.10 - 2.90
	2014	1.98 ± 0.07	2.67 ± 0.03	2.43 ± 0.15	0.16	-0.36	2.10 - 2.85
WLR(%)	2013	34.45 ± 0.65	55.82 ± 1.79	62.05 ± 4.47	0.79	0.70	51.50 - 74.60
	2014	32.12 ± 1.99	54.50 ± 1.51	63.12 ± 4.71	-0.31	-0.21	48.00 - 74.15
CGP(%)	2013	5.16 ± 0.50	7.38 ± 0.71	10.75 ± 9.42	1.27	1.30	0.10 - 41.60
	2014	5.79 ± 0.28	7.58 ± 0.50	11.67 ± 10.75	1.47	1.60	0.20 - 46.55
CD(%)	2013	2.99 ± 0.13	3.48 ± 0.19	6.35 ± 5.91	1.51	2.44	0.10 - 28.60
	2014	3.12 ± 0.10	3.73 ± 0.27	7.41 ± 7.46	1.68	2.46	0.10 - 34.00
CA	2013	5.60 ± 0.26	7.00 ± 0.26	5.83 ± 1.03	0.32	0.63	1.08 - 6.87
	2014	5.66 ± 0.21	7.40 ± 0.17	6.29 ± 0.82	-1.01	2.38	3.26 - 8.47
CTS	2013	5.43 ± 0.15	7.43 ± 0.21	6.41 ± 0.73	-0.51	0.66	4.20 - 8.43

	2014	5.70 ± 0.10	8.00 ± 0.10	6.62 ± 1.02	-0.26	0.57	3.59 - 8.91
CCS	2013	61.10 ± 2.11	77.93 ± 2.35	56.32 ± 6.75	0.76	1.14	36.88 - 83.93
	2014	65.03 ± 1.26	77.00 ± 2.16	57.55 ± 6.08	-0.54	0.89	36.63 - 76.07
PKV	2013	2168.33 ± 169.00	2673.67 ± 94.87	2687.35 ± 393.80	-0.15	-0.16	1602.00 - 3696.00
	2014	2565.66 ± 64.53	2815.33 ± 103.69	3032.00 ± 398.30	-0.65	0.22	1884.00 - 3930.00
HPV	2013	1675.33 ± 100.06	1893.02 ± 143.85	1977.15 ± 256.70	0.78	1.98	1360.00 - 2958.50
	2014	1858.33 ± 102.04	2106.00 ± 138.50	2211.40 ± 300.60	-0.07	0.18	1431.67 - 2975.33
BDV	2013	635.33 ± 43.14	877.67 ± 41.97	721.90 ± 235.70	0.37	0.78	194.00 - 1663.00
	2014	569.33 ± 31.89	817.67 ± 67.72	866.90 ± 202.90	0.05	0.84	358.00 - 1464.67
CPV	2013	3372.00 ± 44.03	3170.33 ± 54.45	3373.00 ± 386.80	0.34	1.25	2429.50 - 4681.50
	2014	3440.33 ± 107.34	3185.00 ± 118.05	3468.00 ± 378.30	-0.24	0.66	2114.50 - 4502.00
SBV	2013	1513.33 ± 43.02	1368.67 ± 84.91	1394.00 ± 227.70	-0.74	1.78	654.00 - 1920.00
	2014	1625.67 ± 71.51	1323.33 ± 58.32	1299.00 ± 216.60	0.13	0.50	775.00 - 1898.67
PeT	2013	6.19 ± 0.02	6.04 ± 0.03	6.25 ± 0.16	0.77	1.18	5.84 - 6.73
	2014	6.31 ± 0.04	6.21 ± 0.04	6.36 ± 0.16	-0.38	0.04	5.80 - 6.73
PaT	2013	82.98 ± 0.54	85.45 ± 1.02	82.91 ± 5.10	-0.55	-0.08	67.80 - 93.10
	2014	83.12 ± 0.10	84.11 ± 0.40	78.59 ± 6.49	0.19	-1.00	62.43 - 94.05

S2. Loadings, Proportion of variance (PV), eigenvalues (E) and Cumulative proportion of variance (CCPV) of top 8 principal components.

Index	Principal components							
	PC1	PC2	PC3	PC4	PC5	PC6	PC7	PC8
BRR	0.055	-0.159	0.619	-0.379	0.242	-0.212	-0.022	-0.226
MRR	-0.020	-0.097	0.715	-0.344	0.193	-0.120	-0.007	0.067
HRR	0.209	-0.182	0.136	-0.527	0.074	0.538	0.124	-0.381
MRL	0.356	0.217	0.386	-0.209	-0.357	-0.262	-0.110	0.369
MRW	0.150	-0.432	0.521	0.010	0.282	0.047	0.307	0.209
WLR	-0.271	-0.257	-0.042	0.535	0.500	-0.257	0.267	0.150
CGP	-0.197	-0.764	0.262	0.403	-0.235	0.167	-0.166	-0.005
CD	-0.221	-0.740	0.238	0.438	-0.240	0.160	-0.182	-0.021
CA	-0.742	-0.174	-0.432	-0.075	0.004	-0.101	0.029	-0.151
CCS	-0.469	-0.305	-0.127	-0.231	0.026	-0.459	0.020	-0.426
CTS	-0.162	-0.155	-0.094	-0.170	-0.439	0.045	0.793	0.103
PKV	-0.851	0.125	0.181	-0.236	-0.036	0.141	-0.055	0.134
HPV	-0.825	0.371	0.281	0.040	0.028	0.179	-0.028	0.109
BDV	-0.592	-0.265	-0.064	-0.470	-0.212	-0.016	-0.151	0.133
CPV	-0.542	0.496	0.519	0.278	-0.166	0.035	0.040	-0.128
SBV	0.151	0.323	0.422	0.451	-0.365	-0.230	0.126	-0.410
PeT	-0.627	0.399	0.063	0.251	0.276	0.236	0.072	-0.045
PaT	0.597	0.128	0.066	0.202	0.075	0.266	0.027	-0.138
E	3.976	2.396	2.243	1.974	1.153	0.995	0.934	0.871
PV %	22.09	13.31	12.46	10.97	6.41	5.53	5.19	4.84
CPV %	22.09	35.40	47.86	58.83	65.24	70.77	75.96	80.80

S3. Identification of QTLs for 94 rice quality traits and estimation of their genetic parameters in RILs populations in 2013 and 2014.

Trait	QTL	Chr.	Marker	LOD Value		PVE%		Additive effect	
				2013	2014	2013	2014	2013	2014
Brown rice recovery	*qBRR2*	2	RM475-RM262	2.51		6.99		-0.86	
	qBRR5	5	RM413-RM18062	2.23		8.98		-0.98	
	qBRR7	7	RM5055-RM82	2.31		6.61		-0.94	
	qBRR11	11	PSM173- PSM175		2.22		8.40		-1.05
	qBRR12	12	PSM180-RI05559		2.88		10.92		-1.17
Milled rice recovery	*qMRR3a*	3	STS4-RM22		2.52		8.30		-1.87
	qMRR3b	3	STS6-STS7		2.05		6.77		-1.69
	qMRR3c	3	OSR13-RM6676		2.01		8.67		-2.09
	qMRR7a	7	RM5055-RM82	2.03		7.99		-2.89	
	qMRR7b	7	RM6835-RM455	2.12		7.46		2.51	
	qMRR8	8	RM407-RM6356	2.33		13.07		3.29	
	qMRR9	9	RM257-RM215	2.20		9.79		2.85	
Head rice recovery	*qHRR1*	1	RM8097-RM212	2.78	4.73	15.56	11.53	4.73	4.96
	qHRR2	2	RM497-RI03718	2.11	2.41	6.23	12.11	-2.92	-5.05
	qHRR3	3	STS6-STS7	2.04	2.12	5.91	4.79	-2.81	-3.19
	qHRR7a	7	RIO5304-RM1186	2.62		12.00		-4.02	
	qHRR7b	7	RM5508-RM8261		2.84		6.92		-3.82
	qHRR9	9	RM7424- RM257	4.38	4.12	15.25	12.70	-4.61	-5.26
Milled rice length	*qMRL1*	1	RM3738-RM1361		7.07		17.89		0.14
	qMRL2	2	RIO04587-RM138	2.52		11.53		-0.11	
	qMRL3a	3	STS6-STS7		3.20		7.76		-0.09
	qMRL3b	3	PSM380-RM135		2.20		5.18		-0.08
	qMRL6	6	RIO4969-RM176	2.93		8.03		0.10	
	qMRL7	7	RIO5304-RM1186		3.17		8.15		-0.10
	qMRL9	9	RM566-RM3700	2.85	2.93	8.92	7.21	-0.10	-0.09
	qMRL11	11	STS21-STS22	2.16		7.66		-0.09	
Milled rice width	*qMRW1*	1	RM3738-RM1361		3.91		8.02		0.04
	qMRW2a	2	RM497-RIO3718		3.52		7.35		-0.04
	qMRW2b	2	RM240-RIO4587		2.21		4.08		0.03
	qMRW5a	5	RM413-RM18062	3.07	11.00	5.76	32.59	0.03	0.08
	qMRW5b	5	RM159-RM413	4.95	5.21	21.65	12.23	-0.06	-0.05
	qMRW9	9	RM566-RM3700		4.08		7.42		-0.04
Width length rate	*qWLR1*	1	RM3738-RM1361		2.07		5.43		-1.10
	qWLR3	3	STS6-STS7		3.67		10.84		1.56
	qWLR5	5	RM413-RM18062		3.71		11.53		1.60
	qWLR6	6	RIO4969-RM176		2.53		35.26		2.79
	qWLR9a	9	RM566-RM3700	3.95		11.42		-1.58	
	qWLR9b	9	RM257-RM215	2.12		5.08		-1.05	
	qWLR11	11	STS22-STS23	3.32		10.33		1.50	
Chalky grain percentage	*qCGP1*	1	RM8110-RM283	2.55		9.80		3.05	
	qCGP5	5	RM413-RM18062	3.97	4.60	12.89	14.87	3.51	4.46
	qCGP8	8	RM544-RM25		1.92		6.89		-3.05
	qCGP9	9	RM257-RM215		2.17		7.38		3.13
Chalkiness degree	*qCD1*	1	RM8110-RM283	2.74		10.40		1.90	
	qCD5	5	RM413- RM18062	3.77	4.75	12.25	15.27	2.07	2.92
	qCD9	9	RM257-RM215		2.15		7.98		2.10

Cooked appearance	*qCA1*	1	STS1-STS2	2.84		11.59		-0.35	
	qCA3	3	STS9-OSR13	2.00		6.39		0.28	
	qCA6	6	RM589-RM527		2.91		12.74		-0.36
	qCA7	7	RM6835-RM455		2.70		8.76		-0.24
	qCA11	11	RM4-PSM173	2.21		7.99		-0.29	
Cooked taste score	*qCTS7*	7	RM5508-RM8261	1.95		6.26		-1.52	
Cooked comprehensive score	*qCCS6*	6	RM589-RM527	2.16	3.03	26.03	11.91	-6.36	-0.36
	qCCS7	7	RM6835-RM455		3.32		11.09		-3.08
	qCCS9	9	RM566-RM3700	2.00		6.68		-2.75	
Peak paste viscosity	*qPKV7a*	7	RM6835-RM455	3.54	2.33	5.41	9.22	-154.00	-118.90
	qPKV7b	7	RI03938-RM3555	2.10		3.22		-116.50	
	qPKV8	8	RM407-RM6356	3.01	2.77	11.33	9.20	126.59	116.17
Hot paste viscosity	*qHPV7*	7	RM6835-RM455	2.21		3.51		-97.11	
	qHPV3a	3	RM22-STS5		3.35		10.26		-98.49
	qHPV3b	3	RM227-RM55		2.18		6.25		-76.97
	qHPV4	4	RS3-RS4		2.08		-11.48		103.86
	qHPV8	8	RM407-RM6356		2.63		6.69		79.32
	qHPV9	9	RM7424-RM257		3.41		11.89		107.13
Breakdown viscosity	*qBDV7*	7	RM6835-RM455	3.80	2.21	11.84	7.87	-82.66	-57.98
Cool paste viscosity	*qCPV3*	3	RM22-STS5		4.21		12.54		-133.93
	qCPV9	9	RM7424-RM257		3.03		10.12		121.66
Setback viscosity	*qSBV1a*	1	RM6902-RM1167	2.16		7.65		66.61	
	qSBV1b	1	RM212-RM3738		2.95		10.43		-63.05
	qSBV8a	8	RM152-RM544	2.99		13.78		-90.43	
	qSBV8b	8	RI03187-RM5556		2.38		12.39		-68.68
Peak time	*qPeT3*	3	RM22-STS5		2.28		7.45		-0.04
	qPeT7	7	RM6835-RM455		2.08		10.62		-0.05
	qPeT9	9	RM7424-RM257		2.00		7.42		0.05
	qPeT11	11	STS21-STS22		2.18		8.06		0.05
Pasting temperature	*qPaT3*	3	RM135-R3M53	2.50		6.27		0.99	
	qPaT4	4	RM307-RM4833		2.07		11.85		2.22
	qPaT6	6	RI04969-RM176		2.50		11.78		-2.31
Principal component	*qPC1-7*	7	RM6835-RM455		4.79		16.62		-0.77
	qPC2-1	1	RM3738-RM1361		4.06		11.13		-0.54
	qPC2-5	5	RM413-RM18062		3.21		8.26		-0.47
	qPC2-9	9	RM3700-RM7424	4.25	3.24	15.40	12.21	0.58	0.57
	qPC3-5	5	RM413-RM18062	2.94	4.17	14.85	11.58	0.54	0.46
	qPC3-9	9	RM566-RM3700		3.02		8.73		-0.41
	qPC4-2	2	RM497-RI03718		3.82		16.62		0.56
	qPC4-3	3	RM407-RM6356		3.34		10.63		0.45
	qPC4-5	5	RM159-RM413		2.68		7.58		-0.38
	qPC4-6	6	RI04969-RM176	3.41		9.50		-0.42	
	qPC4-7	7	RI05304-RM1186	3.36		10.92		0.45	
	qPC6-3a	3	STS6-STS7	3.68		10.85		-0.36	
	qPC6-3b	3	OSR13-RM6676	2.86		15.29		-0.46	
	qPC6-9	9	RM566-RM3700	3.22		15.51		0.42	

qPC8-4	4	R307-RM4833	5.18	14.04	0.36
qPC8-11	11	STS20-STS21	2.89	8.65	0.29

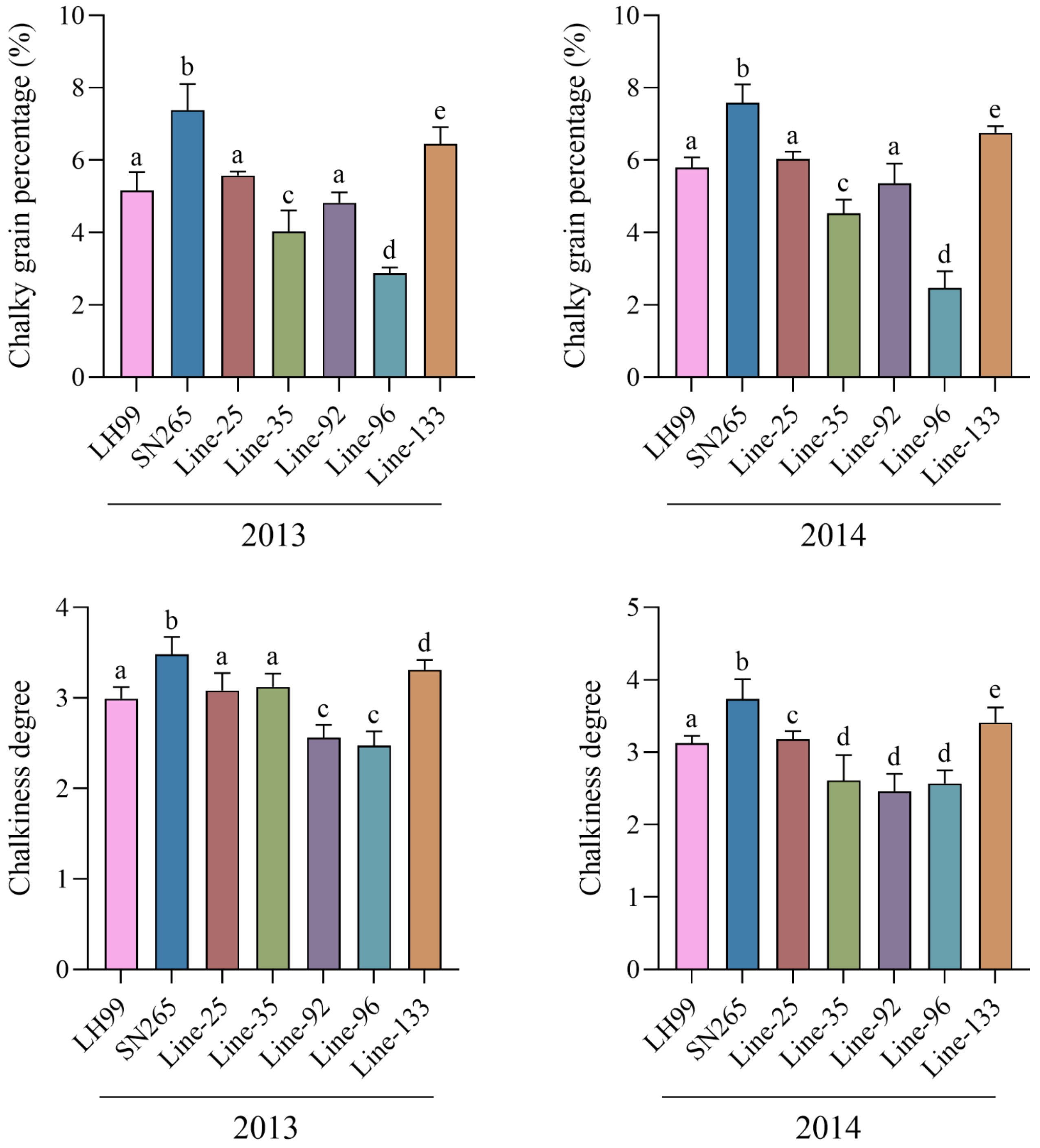

S4. The comparison between the excellent lines and the parents was in chalky grain percentage and chalkiness degree. Different letters represent significant at P < 5% (Student's t-test).
